# Comparison of the Radiopacities of Different Root-End Filling and Repair Materials

**DOI:** 10.1155/2013/594950

**Published:** 2013-10-23

**Authors:** Jale Tanalp, Meriç Karapınar-Kazandağ, Semanur Dölekoğlu, Mehmet Baybora Kayahan

**Affiliations:** ^1^Department of Endodontics, Faculty of Dentistry, Yeditepe University, Bagdat Caddesi 238, Göztepe, 34728 Istanbul, Turkey; ^2^Department of Oral Radiology, Faculty of Dentistry, Yeditepe University, Istanbul, Turkey

## Abstract

This study evaluated the radiopacity of 3 repair materials, Biodentine, MM-MTA, and MTA Angelus. Standardized cylindrical rings were prepared. Samples of Biodentine MM-MTA and MTA Angelus were prepared (*n* = 10 in each group), filled into the rings, and preserved at 37°C until setting. A 1 mm thick dentin slice was used as control. All set specimens were removed and radiographed along with the dentine slice and a graduated aluminium step wedge. Digital images were transferred to the computer using a software. The radiographic densities of the specimens were determined, and the values were converted into millimetres of aluminium (mm Al). One-way ANOVA was used for intergroup comparison, whereas Tukey HSD test was used for detecting the group with the difference. The mean radiopacities of Biodentine, MTA Angelus, and MM-MTA were 2.8 ± 0.48, 4.72 ± 0.45, and 5.18 ± 0.51 mm Al, respectively. The radiopacity of Biodentine was significantly lower compared to other materials (*P* = 0.001), whereas no significant difference was noted between MTA Angelus and MM-MTA (*P* = 0.109). All materials had significantly higher radiopacities compared to dentine. The relatively lower radiopacity of Biodentine can be improved to achieve more reliable results in procedures such as retrograde fillings.

## 1. Introduction

During orthograde endodontic treatment, unsuccessful results can be obtained, such as the persistence of apical infection. In case an endodontic treatment fails, the dental practitioner has the option of an apical surgical intervention, which generally includes the resection of the apical root portion associated with periapical infection [1]. Following apical surgery, the necessity arises for obturating the resected area with a retrograde filling material that thoroughly isolates the root canal system from the surrounding tissues in order to promote healing. The necessity of a thorough and hermetic obturation is not only valid for retrograde fillings, but a complete and successful isolation is a prerequisite of many interventions such as closure of root perforations and pulp capping procedures. Consequently, the material to be selected for retrograde fillings as well as other sealing and repair procedures should exhibit favorable characteristics such as good biocompatibility, sealing ability, minimal leakage, low cytotoxicity, and lack of dissolution in tissue fluids. Mineral trioxide aggregate is a material that specifically gained attention in recent years because it fulfils many of the prerequisites expected from a material intended to be used as a retrograde filling, pulp capping, or repair material [2, 3]. 

Another important feature expected from an ideal repair material is sufficient radiopacity in order to be easily discerned from anatomical structures [1, 4]. This is of specific importance when retrograde fillings are concerned which are applied in relatively lower thicknesses compared to regular root filling materials (gutta-percha + sealer). The radiograph taken following apicoectomy and retrograde filling placement must ensure adequate apical sealing so that the practitioner can complete the surgical procedure and this is only possible by selecting a material that favorably reveals its presence in radiographs. It has been emphasized that in a radiograph taken postoperatively, it must be confirmed that the material is within the retrograde cavity, well packed without any defects or voids, easily discerned from the dentine tissue, and superimposed bone trabeculae [5]. 

There have been a variety of filling materials tested for their ability to serve as retrograde fillings. Amalgam, composite resins, ethoxybenzoic acid cements, Cavit, and glass ionomer cements are some examples of materials that were preferred as retrograde fillings [6]. Mineral trioxide aggregate (MTA), first introduced in 1993, has proven to be a reliable material not only as a retrograde filling but also in procedures such as pulp capping, pulpotomy, closure of perforations, and single-visit apexification. MTA has basically been derived from Portland cement, with the inclusion of some additives such as Bismuth oxide. Its low cytotoxicity and solubility, biocompatibility, induction of cementogenesis, and bone formation, setting in the presence of blood or moist environment and good sealing ability, have rendered MTA to be a material of choice, specifically in cases where there is direct contact with connective tissue. Its alkaline pH and calcium ion release are additional advantages [7]. White MTA (MTA Angelus) (Angelus, Londrina, Brazil) is a commonly used form of mineral trioxide aggregate, overcoming the disadvantages of gray MTA which has been shown to possess some adverse properties such as staining of tissues. It also contains Bismuth oxide as a radiopacifier. 

Recently, there have been attempts to produce alternative materials to original mineral trioxide aggregate. To serve this purpose, new materials were produced that contain tricalcium silicate and that have the potential to release calcium hydroxide in solution, which, when in contact with tissue fluids, forms hydroxyapatite [8]. Tricalcium silicate cement has been indicated to have comparable physical and chemical properties to mineral trioxide aggregate [9, 10]. In addition, it has been stated that zirconium oxide is used as an alternative radiopacifier with some calcium silicate-based cements resulting in optimal properties [9, 11]. 

 Biodentine (Septodont, Saint Maur des Fossés, France), one of these innovative materials, is primarily composed of tricalcium silicate, whereas zirconium oxide is added as a radiopacifier. Biodentine powder also contains calcium carbonate, while the liquid consists of calcium chloride, and a hydrosoluble polymer. Analysis of the powder and liquid using X-ray fluorescence spectroscopy (XRF) displayed the presence of calcium, silicon, and zirconium oxide in the powder and sodium, magnesium, chloride and calcium together with the water in the liquid phase [12]. Laurent et al. [13] suggested that Biodentine can be used as a restorative material in addition to other endodontic indications. Laurent et al. [14] postulated that when Biodentine was applied directly onto the pulp, it induced an early form of reparative dentine synthesis.

MM-MTA (Micro Mega, Besançon, France) is also a retrograde MTA-containing material launched in capsule form to be used after mixing by a high frequency mixer. One unique characteristics of the product, according to the manufacturer, is the addition of CaCO_3_, which reduces working time, eliminating one of the disadvantages of the original MTA. According to the manufacturer, the capsule form not only causes a cost-effective application but also facilitates the formation of a homogeneous mixture that is difficult to achieve by hand instruments.

As these three abovementioned materials are recommended to be used as retrograde fillings, their radiopacity is an important factor in their selection, among other expected qualities. 

The ISO 6876:2001 has established 3 mm Al as the minimum radiopacity value for endodontic cements [15]. Furthermore, according to ANSI/ADAspecification number 57, all endodontic sealers should be at least 2 mm Al more radiopaque than dentin or bone [16]. A generally used methodology for the evaluation of radiopacity of filling materials is the one developed by Tagger and Katz where radiographic images of the material are taken alongside an aluminium step-wedge (penetrometer). The radiographic images are digitized, and different gray-scale values are detected by specific software [17].

A survey of the literature reveals that studies pertaining to the radiopacity of Biodentine and MM-MTA are rather limited. The purpose of this study was to comparatively evaluate the radiopacity values of 3 root-end filling materials, Biodentine and MM-MTA and MTA Angelus. The hyphothesis was that the evaluated materials had radiopacities above dentin and equal or above the established ISO standards.

## 2. Materials and Methods

Cylindrical stainless steel rings 10 mm in diameter and 1 mm in thickness were prepared to obtain uniform specimens from each group compatible with ISO 6876 specifications [15]. Biodentine, MM-MTA, and MTA Angelus were prepared according to the manufacturers' instructions, filled into the rings (*n* = 10), and preserved at 37°C until they set. A 1 mm thick dentin slice was used as control. All set specimens were removed from the rings, and the homogeneity of thicknesses was tested by a digital caliper (Mitutoyo Corp, Tokyo, Japan). The dentin cylinder to be used as control was obtained by cutting the roots of caries-free freshly extracted human teeth in a 1 mm thick section. The thickness of the dentine slice was also checked by a caliper. 

The specimens were placed on storage phosphor plate (Digora, Soredex, Helsinki, Finland) and radiographed along with the dentine slice and a graduated 99.5% pure aluminium step-wedge with 1 mm increments using a Trophy (Novalix, Croissy-Beaubourg, France) intraoral X-ray unit operating at 65 kV and 8 mA, with a film-focus distance of 30 cm. Storage phosphor plates were scanned using Digora Optime Scanner (Soredex, Helsinki, Finland). Digital images were converted to the computer using Digora for Windows software (Soredex, Helsinki, Finland). The radiographic densities of the specimens were determined using the toolbox of the same software, and the values were converted into millimeters of aluminium (mm Al) and recorded. Three exposures were made for each sample, and density measurements were made at 5 separate points of the samples. The average of the measured values was taken for each sample. 

Statistical package for social sciences (SPSS) for Windows 15.0 program was used for statistical analysis. Intergroup comparison of parameters was performed using One-way ANOVA test, whereas Tukey HDS test was used for the detection of the group causing the difference. Student *t*-test was used for the comparison of parameters between 2 groups. Significance level was set at *P* < 0.05.

## 3. Results

All materials tested had significantly higher radiopacities compared to the control (dentin) in their respective groups (*P* < 0.001).  Table 1 represents the average radiopacity values obtained for each group. The average radiopacity of the Biodentine group was significantly lower compared to the MTA Angelus and MM-MTA groups (*P* < 0.01). No statistically significant difference was observed between the radiopacities of MTA Angelus and MM-MTA (*P* > 0.05). 

## 4. Discussion

The methodology developed by Tagger and Katz [17] was used in the present study where the radiopacity of materials is calculated using standardized samples radiographed next to an aluminum step wedge. The radiographs are then digitized, and the specimens' radiopacity is compared to that of the aluminum step wedge using computer software. This methodology has been shown to determine the radiopacity of the tested materials in a simple and easily reproducible manner with reliable outcomes [18]. 

Root-end filling materials should be distinguishable from the adjacent bone and root dentin. As there is no international standardization for establishing the minimum acceptable radiopacity values for materials intended to be used as root-end fillings [1, 4], the 3 mm Al threshold to be distinguishable from the adjacent bone and root dentin was used during interpretation of differences.

In the present study, both MTA Angelus and MM-MTA yielded radiopacity values that exceeded the minimum set standard (3 mm Al). MTA Angelus was assessed in other studies pertaining to radiopacity, and varying results were obtained by different researchers. Vivan et al. [19] found this material to have an average radiopacity as high as 6.45 mmAl, whereas lower results were obtained by others [18]. Methodological variations such as the exposure voltage, film-focus distance, and the preparation of samples may be some reasons for the inconsistency between the results. The powder : liquid mixing ratio of the material may also influence the radiopacity characteristics. Although a 3 : 1 powder to liquid ratio is advocated by the manufacturer, other mixture types are also possible that might change the radiopacity value. A recent study concluded that higher radiopacity values can be obtained when a 4 : 1 powder to liquid ratio was selected for White MTA [20]. Although such a preparation might be favorable in cases where a high radiopacity is crucial, the degree to which the physical and biological characteristics of the material are affected by such a strategy is questionable. Nevertheless, it has been suggested that the powder to liquid ratio should be strictly followed in order not to compromise the overall quality of the retrograde filling. 

Bismuth oxide (Bi_2_O_3_) is added to the formulation of MTA Angelus for the provision of radiopacity. Concerns have been expressed by authors regarding bismuth oxide since it does not partake in the setting reaction [21]. Some studies also showed that Bi_2_O_3_ is toxic toward human dental pulp cells [22]. Coomaraswamy et al. [23] demonstrated that the addition of Bi_2_O_3_ to Portland cement (PC) dramatically changes the physical properties of the material, producing flaws within the cement matrix and increasing the porosity by leaving more unreacted water. These findings may result in higher rates of solubility and degradation. 

Biodentine is a retrograde and repair material launched in capsule form consisting of a di- and tricalcium silicate powder which is mixed with aqueous calcium chloride solution. This material uses zirconium oxide to give radiopacity. Contrary to bismuth oxide, zirconium oxide was shown to possess biocompatible characteristics and indicated as a bioinert material with high mechanical properties and favorable resistance to corrosion [24]. Surprisingly, Biodentine not only yielded a lower radiopacity value compared to the other materials, but the average radiopacity for this material was slightly lower than the standard set by ISO, which is rather an undesirable property for a retrograde material. However, comments on this result should be made with caution as there is very limited information on this new material. On the other hand, a clinical observation indicated that the radiopacity of Biodentine is in the region of dentin and the cement is not adequately visible in the radiograph, resulting in difficulty in correct application [25]. Though this statement is made on a subjective basis and is dependent only on visual observation, it is nevertheless supportive of the results of the present study in terms of the inadequacy of the radiopacity of this repair material. Though different results may be obtained under different study settings, if confirmed by further research, suggestions can be brought to enhance the overall radiopacity of this material that has been otherwise shown to be convenient, efficient, and well- tolerated dentine substitute [26].

MM-MTA, another one of the tested materials in the present study was recently launched to the market. To the authors' knowledge, there is yet (May 2013) no study regarding the physical properties of this material, including radiopacity. According to the manufacturer, the inclusion of calcium carbonate significantly increases its physical characteristics and reduces setting time that has been observed as a drawback of conventional MTA. Furthermore, bismuth oxide is the radiopacifier used in this product. Though MM-MTA yielded the highest radiopacity value in the present study, this property should be further supported by other advantages to make this material a feasible option in retrograde and repair procedures.

Within the limitations of the present study, though all materials had higher radiopacities compared to dentine, the relatively lower radiopacity of Biodentine compared to other materials can be improved to achieve more reliable results in procedures such as retrograde fillings. Radiopacity is only one component of an overall successful treatment procedure and multiple factors need to be considered while selecting the most appropriate material. Further research that focuses on the radiopacity as well as other physical and biological characteristics of these materials will definitely be complementary in providing the practitioner with a clearer picture regarding their general quality and suitability for retrograde and repair procedures. 

## Figures and Tables

**Table 1:** Average radiopacity and SD associated with each tested material and comparison of different materials.

	Radiopacity	^ +^ *P*
	Mean ± SD	
Biodentine	2,80 ± 0,48	0,001**
Angelus	4,72 ± 0,45	
MMTA	5,18 ± 0,51	

Biodentine/Angelus ^++^ *P*	0,001**	
Biodentine/MMTA ^++^ *P*	0,001**	
Angelus/MMTA ^++^ *P*	0,109	

^+^Oneway ANOVA test.

^++^Tukey HSD test.

**Statistically significant.
